# Authors' Reply: Aligning Noninferiority Assumptions and Decision Rules in a Protocol for a Study on Adjunctive Acupuncture for Late-Life Depression

**DOI:** 10.2196/94790

**Published:** 2026-05-01

**Authors:** Qingnan Fu, Kaihui Xiao, Jie Zhang, Yang Li, Yuxian Wang, Molin Jiang, Zengqi Man, Jing Yang, Wei Lu

**Affiliations:** 1 Department of Psychosomatic Medicine, Beijing Traditional Chinese Medicine Hospital Affiliated to Capital Medical University, 23 Back Street of Art Museum, Dongcheng District, Beijing, China, 86 87906524; 2 Neurology Ward 1, Dongzhimen Hospital, Beijing University of Chinese, Beijing, China; 3 Acupuncture and Massage College, Capital Medical University, Beijing, China

**Keywords:** depression, mild to moderate depression in older people, acupuncture, randomized controlled trial, protocol

We thank the author of the letter to the editor [1] for their keen interest in our study protocol [2] and for raising highly relevant methodological considerations regarding noninferiority trial designs. We agree that transparent alignment between assumptions, end points, and analytical rules is essential, particularly for complex adjunctive interventions in older adults. We welcome this opportunity to clarify and expand upon the statistical framework of our trial.

The author correctly points out that the approximately 30% figure frequently cited from the Sequenced Treatment Alternatives to Relieve Depression (STAR*D) trial [3] typically refers to the remission rate, whereas our primary end point is the response rate (≥50% reduction in 17-item Hamilton Depression Rating Scale [HAMD-17] score). We appreciate this distinction. Our rationale for adopting a conservative baseline assumption of a 30% response rate for the control group is rooted in the specific demographics of our study: people with late-life depression.

Evidence consistently demonstrates that older adults exhibit lower and slower response trajectories to selective serotonin reuptake inhibitor monotherapy (such as with citalopram) compared to the general adult populations studied in STAR*D [4]. Therefore, anticipating a 30% to 35% response rate in this specific older cohort provides a conservative and clinically realistic baseline for our power calculations. Nonetheless, to address the sensitivity to plausible response-rate assumptions, our statistical analysis plan will include a sensitivity analysis calculating the actual statistical power post hoc based on the observed pooled response rate, ensuring the adequacy of the prespecified margin.

To provide absolute clarity on our inferential conventions, the noninferiority of acupuncture plus citalopram versus citalopram alone will be evaluated using the 2-sided 95% CI of the difference in response rates between the two groups (intervention minus control). Consistent with CONSORT (Consolidated Standards of Reporting Trials) guidelines for noninferiority trials, noninferiority will be declared if the lower bound of this 95% CI is greater than our prespecified margin of –15%. The α level for this analysis is set at .05 (2-sided), which aligns mathematically with the parameters used in our sample size planning.

We fully agree that conclusions in noninferiority trials are highly sensitive to the analysis population and missing data handling [5]. To prevent the bias toward the null often associated with intention-to-treat analyses in noninferiority designs, our primary conclusion will require noninferiority to be demonstrated in both the full analysis set and the per-protocol set.

Regarding missing data, we will use a mixed-effects model for repeated measures for continuous secondary outcomes and multiple imputation for the binary primary end point under the missing at random assumption. Additionally, tipping-point sensitivity analyses will be conducted to assess the robustness of our conclusions against potential missing not at random mechanisms, which is especially important given the unequal contact time between the two arms.

We are grateful to the author for prompting this methodological dialogue. These clarifications will be formally embedded into our final statistical analysis plan prior to database lock, ensuring that the eventual trial results are robust, transparent, and accurately interpretable.

## Abbreviations

CONSORT: Consolidated Standards of Reporting Trials
HAMD-17: 17-item Hamilton Depression Rating Scale
STAR*D: Sequenced Treatment Alternatives to Relieve Depression
